# Real world data on young patients with high-risk diffuse large B-cell lymphoma treated with R-CHOP or R-CHOEP - MYC, BCL2 and BCL6 as prognostic biomarkers

**DOI:** 10.1371/journal.pone.0186983

**Published:** 2017-10-31

**Authors:** Mette Ølgod Pedersen, Anne Ortved Gang, Peter Brown, Michael Pedersen, Helle Knudsen, Signe Ledou Nielsen, Tim Poulsen, Tobias Wirenfeldt Klausen, Estrid Høgdall, Peter Nørgaard

**Affiliations:** 1 Dept. of Pathology, Herlev and Gentofte Hospital, Herlev, Denmark; 2 Dept. of Hematology, Herlev and Gentofte Hospital, Herlev, Denmark; 3 Dept. of Hematology, Rigshospitalet, Copenhagen, Denmark; Cornell University, UNITED STATES

## Abstract

**Background:**

Double expression of MYC and BCL2 proteins (DE) and double-hit *MYC+BCL2/BCL6* translocations (DH) were established as important biomarkers in patients with diffuse large B-cell lymphoma (DLBCL) by the 2016 revision of the World Health Organization classification of lymphoid neoplasms. Whether this applies to the subgroup of young patients with high risk DLBCL is not known. We previously found that in a uniform retrospective population-based cohort of patients aged 18–60 years with high-risk DLBCL, the addition of etoposide to R-CHOP chemotherapy (R-CHOEP) resulted in improved survival mainly in patients with germinal center B-cell like (GCB) immunophenotype. The aim of this study was to investigate the prognostic and predictive value of DE and DH in this patient cohort.

**Methods:**

Data on all young Danish patients diagnosed with de novo high-risk DLBCL 2004–2008 and treated with R-CHOP or R-CHOEP were obtained from the Danish Lymphoma database (n = 159). Tumor samples were available from 103 patients. MYC and BCL2 proteins were analyzed with quantitative immunohistochemistry (IHC) using different cut off values. *MYC-*, *BCL2-* and *BCL6*-translocations were examined with fluorescent in situ hybridization (FISH).

**Results:**

DE with MYC>75% and BCL2>85% was an independent negative prognostic marker of progression free survival (PFS) in patients treated with R-CHOP but not R-CHOEP (p<0.001), also after exclusion of patients with DH. A predictive effect of DE for response (PFS) to R-CHOEP vs. R-CHOP was almost significant (p = 0.07). DH was not prognostic in this patient cohort.

**Conclusion:**

In young patients with high-risk DLBCL, treatment with R-CHOEP may overcome the negative prognostic impact of DE observed in patients treated with R-CHOP.

## Introduction

Young patients with high-risk diffuse large B-cell lymphoma (DLBCL) constitute a subgroup of patients for whom improved prognostication is strongly warranted. The vast majority of studies investigating prognostic markers in DLBCL included elderly patients with mixed clinical features and treated with R-CHOP or R-CHOP-like chemotherapy regimens. In the revised WHO classification from 2016 [1] cell of origin (COO) [2], double-hit (DH) *MYC+BCL2/BCL6* translocations [3] and double expression (DE) of MYC and BCL2 protein [4;5] were pointed out as the currently most established and important biomarkers in DLBCL.

Intensive chemotherapy regimens are often used for younger patients with high-risk DLBCL in attempts to improve outcome from the standard treatment with cyclophosphamide, doxorubicin, vincristine, prednisone and rituximab (R-CHOP). One such alteration is the addition of etoposide to R-CHOP, which is used in R-CHOEP [6–10]and DA-EPOCH-R [11–14]. Reported overall survival (OS) after treatment with R-CHOEP and DA-EPOCH-R varies between 76%–85% and 77%–94% respectively.

No randomized trials have been conducted comparing R-CHOP and R-CHOEP. Therefore we collected a unique, retrospective population based cohort including all young Danish patients diagnosed with high-risk DLBCL from 2004 through 2008 and treated with R-CHOP or R-CHOEP, with the purpose of comparing outcome and responses to treatment in a “real world” setting. In addition we wanted to investigate the prognostic and predictive effects of established biomarkers; cell of origin (COO), double-hit (DH) *MYC+BCL2/BCL6* translocations and double expression (DE) of MYC and BCL2 protein.

We previously published that patients in this cohort treated with R-CHOEP had improved PFS and OS compared to patients treated with R-CHOP [6]. In addition, we found that the improved outcome associated with R-CHOEP was mainly observed in patients with germinal center B-cell like (GCB) immunophenotype [15]. In the current study we aimed to investigate the prognostic value of *MYC*, *BCL2* and *BCL6* gene translocations and MYC and BCL2 protein expression in the same patient cohort. We wanted to investigate whether these markers were also of prognostic value in young patients with high-risk DLBCL and if so, whether the prognostic effect was seen after treatment with both R-CHOP and R-CHOEP. We also wanted to evaluate whether any of the markers could predict response to treatment with R-CHOEP compared with R-CHOP.

## Materials and methods

### Cohort

A population based, previously well described, cohort of 159 young patients with high-risk (age aaIPI 2–3) DLBCL [6] was investigated. The cohort was extracted from the Danish Lyfo Registry [6;16] and included all young (18–60 years) Danish patients diagnosed with de novo high-risk DLBCL between January 2004 and December 2008. Only patients treated with either R-CHOP14 or R-CHOEP14 were included. Detailed cohort inclusion criteria and treatment strategies were previously described [6]. The research was approved by the ethics committees in Denmark (H-3-2009-142) and was conducted according to the principles expressed in the Declaration of Helsinki.

### Pathology review

Collection of H&E stained sections, immunohistochemistry sections and formalin-fixed paraffin embedded (FFPE) tissue blocks was successful for the majority of patients (Sections: n = 133; FFPE tissue blocks: n = 111). Central review was carried out according to the WHO classification [17] as previously described [15]. After a pathology review, 19 patients were excluded (transformed from indolent malignant lymphoma (n = 4), HIV (n = 1), Burkitt Lymphoma (n = 1), insufficient material to confirm diagnosis (n = 5), T-cell/histiocyte-rich B-cell lymphoma (n = 7) and intravascular lymphoma (n = 1).

### Tissue

FFPE tissue blocks were available from 111 patients. In 103 of these there was sufficient tissue for further analysis. Samples with a suitable amount of tissue (n = 34) were used for tissue micro arrays (TMAs) (1 mm cores in duplicate). When the material was sparse, whole tissue-sections were analyzed (n = 69). Two μm sections were cut for supplemental FISH and IHC analyses.

### Fluorescent *in situ* hybridization (FISH)

A total of 103 out of the 159 patients had tissue available for FISH. FISH for detection of *MYC*, *BCL2* and *BCL6* translocation was successful in 99 of 103 patients and performed as previously described [18]. The following FISH probes were used according to the manufacturer’s recommendations: MYC split (ZytoVision, Zyto Light, SPEC CMYC Dual Color Break Apart Probe), BCL2 split (Dako, BCL2 FISH DNA Probe, Split Signal, Code Y5407) and BCL6 split (Dako, BCL6 FISH DNA Probe, Split Signal, Code Y5408).

### Immunohistochemistry (IHC)

IHC for *MYC* protein expression was carried out centralized at the Department of Pathology, Herlev Hospital in accordance with guidelines from routine diagnostic work-up. FFPE tissue sections were pretreated on “PreTreatment Module” (PT-Link, Dako) including deparaffinization, rehydration and epitope retrieval according to the manufacturer’s instructions at high pH (9.0). “Dako Autostainer Link 48” was used for the immunohistochemical analyses. The EnVisionTM FLEX+ (Dako) visualization kit was used in accordance with the manufacturer’s instructions. The monoclonal c-Myc antibody from Epitomics was used (clone y69/EP121), diluted 1:100 in EnVision Flex Antibody Diluent (Dako).

The remaining IHC analyses were carried out both at local departments of pathology throughout Denmark as part of the routine diagnostics as well as at the Department of Pathology at Herlev Hospital as previously described [15].

IHC was successfully carried out for MYC (n = 103), BCL6 (n = 100), BCL2 (n = 103), CD10 (n = 103) and MUM1 (n = 75).

MYC and BCL2 were scored as continuous variables as the percentage of positive tumor cells regardless of the staining intensity. CD10, BCL6 and MUM1 were scored as dichotomous variables, as positive (>30%) and negative (<30%) and tumors were classified as germinal center like (GCB) and non-GCB as previously described by Hans et al. [19].

Scoring of MYC protein expression was carried out independently and blinded by PN and MOP. Kappa statistics showed very good agreement (MYC>75%, kappa value: 0.83; MYC>40%, kappa value: 0.82). The remaining IHC sections were scored by a haematopathologist (PN, HK, AF, SLN) and MOP as previously described [15]. In cases with a discrepancy of more than 20% in continuous variables, consensus was obtained by microscopy with a multi-headed microscope. With a discrepancy below 20%, the score from the experienced haematopathologist was used. In cases with a discrepancy in dichotomous variables, consensus was obtained by microscopy with a multi-headed microscope.

### Statistics

Chi-square test/Fisher’s exact test and the Mann-Whitney test/Kruskal Wallis tests were used when categorical and continuous variables were compared between groups.

The cumulative probability of death (OS) and relapse (PFS) were estimated with the Kaplan-Meier method and the differences in OS and PFS between groups were analyzed using the log-rank test.

Cox proportional hazard models were used to investigate statistical interactions and multivariable analyses and here the Wald test was used. P values <0.05 were regarded as significant. Statistical analyses were carried out using the IBM SPSS Statistics 20 (IBM corp. Armonk, NY, USA) software and R statistical software version 3.2.3 (R Foundation for Statistical Computing, Vienna, Austria).

The web application “Cutoff Finder” [20] was used to determine the optimal cutoff points for MYC and BCL2 protein expression. “Option 5” in which Cox propertional hazard models including dichotomized variables and the outcome variable progression free survival (PFS) was used to determine the most optimal cutoff value as the point with the most significant split (log-rank test)in R-CHOP treated patients.

The primary endpoint was PFS. Secondary endpoints included overall survival (OS) and overall response rate (ORR) as previously described [15;21].

### Funding

This work was supported by the Department of Pathology, Herlev Hospital; Dansk Kræftforsknings Fond and Roche A/S (unrestricted grant).

## Results

### Clinical characteristics

A total of 103 patients were investigated in the study, 63 were treated with R-CHOP and 40 with R-CHOEP. There was no difference in baseline clinical characteristics between the groups except that patients treated with R-CHOEP were slightly younger (Table 1).

**Table 1:** Baseline clinical characteristics and treatment response in all patients and in patients treated with R-CHOP and R-CHOEP.

		R-CHOP (n = 63)		R-CHOEP (n = 40)		TOTAL (n = 103)		p*
		n	*%*	n	%	n	%	
Sex	F	24	*38*	21	53	45	44	0.2
	M	39	*62*	19	48	58	56	
Ann Arbor stage	1	1	*2*	0	0	1	1	0.6
	2	2	*3*	3	8	5	5	
	3	26	*41*	17	43	43	42	
	4	32	*51*	20	50	52	50	
Performance status	0–1	43	*68*	27	68	70	68	0.9
	2–4	20	*32*	13	33	33	32	
aaIPI	2	49	*78*	31	78	80	78	1.0
	3	14	*22*	9	23	23	22	
LDH	Elevated	60	*95*	39	98	99	96	0.6
	not elevated	3	*5*	1	3	4	4	
Age ≤ 55		36	*57*	32	80	68	66	0.02
Age > 55		27	*43*	8	20	35	34	
Response evaluation	CR, Cru, PR	47	*75*	35	88	82	80	0.3
	SD, PD	9	*14*	3	8	12	12	
	dead before evaluation	5	*8*	1	3	6	6	

R-CHOP, rituximab, cyclophosphamide, doxorubicin, vincristine, prednisone; R-CHOEP, rituximab, cyclophosphamide, doxorubicin, vincristine, etoposide, prednisone; F, female; M, male; aaIPI, age-adjusted international prognostic index; CR, complete remission; CRu, CR unconfirmed; PR, partial remission; SD, stable disease; PD, progressive disease; LDH, lactate dehydrogenase.

* *p* -Value: comparing R-CHOP to R-CHOEP.

Patients treated with R-CHOEP had significantly improved outcome when compared to patients treated with R-CHOP with regards to PFS (HR 0.44; 95%CI: 0.22–0.87; p = 0.02). This finding was not statistically significant regarding OS (HR 0.57; 95%CI: 0.26–1.2; p = 0.16). Gender and aaIPI were prognostic factors in the total cohort and in R-CHOP treated patients but not in patients treated with R-CHOEP. Age>55 was a negative prognostic factor in R-CHOEP but not in R-CHOP treated patients (Table 2).

**Table 2:** Univariate survival analyses in all patients and in patients treated with R-CHOP and R-CHOEP.

		R-CHOP							R-CHOEP							TOTAL						
		PFS				OS			PFS				OS			PFS				OS		
		n	HR	*95% CI*	p	HR	*95% CI*	p	n	HR	*95%*	p	HR	*95%*	p	n	HR	*95%*	p	HR	*95%*	p
SEX	F	24	2.3	*1*.*0–5*.*2*	0.03	3.2	*1*.*1–9*.*5*	0.026	21	1.5	*0*.*47–5*.*0*	0.5	0.98	*0*.*26–3*.*6*	1.0	45	2.2	*1*.*2–4*.*3*	0.01	2.20	*1*.*0–4*.*8*	0.04
	M	39							19							58						
aaIPI	2	49	2.1	*0*.*97–4*.*5*	0.05	3.9	*1*.*7–9*.*2*	0.001	31	0.79	*0*.*17–3*.*7*	0.8	1.0	*0*.*20–4*.*9*	1.0	80	1.5	*0*.*78–3*.*1*	0.2	2.6	*1*.*2–5*.*4*	0.009
	3	14							9							23						
Age	Age ≤ 55	36	0.73	*0*.*36–1*.*5*	0.4	1.1	*0*.*46–2*.*5*	0.9	32	4.9	*1*.*5–16*	0.004	7.5	*2*.*0–28*.*3*	0.001	68	1.4	*0*.*75–2*.*6*	0.3	2.0	*0*.*98–4*.*0*	0.05
	Age > 55	27							8							35						
FISH	*MYC +*	6	*	*	*	*	*	*	7	*	*	*	*	*	*	13	0.60	*0*.*21–1*.*7*	0.3	0.63	*0*.*19–2*.*1*	0.4
	*MYC -*	55							31							86						
	*BCL6 +*	17	1.3	*0*.*62–2*.*8*	0.5	1.6	*0*.*68–3*.*9*	0.3	10	1.8	*0*.*53–6*.*2*	0.3	1.6	*0*.*40–6*.*4*	0.5	27	1.4	*0*.*77–2*.*4*	0.3	1.6	*0*.*79–3*.*4*	0.2
	*BCL6 -*	44							28							72						
	*BCL2 +*	17	0.83	*0*.*37–1*.*9*	0.7	0.52	*0*.*18–1*.*5*	0.2	12	0.81	*0*.*22–3*.*1*	0.8	0.59	*0*.*12–2*.*8*	0.5	29	0.8	*0*.*41–1*.*6*	0.5	0.5	*0*.*22–1*.*3*	0.2
	*BCL2 -*	44							26							70						
	DH MYC + BCL2/BCL6 +	3	*	*	*	*	*	*	5	*	*	*	*	*	*	8	0.50	*0*.*12–2*.*0*	0.4	0.33	*0*.*045–2*.*4*	0.2
IHC	BCL2 > 85%	35	2.7	*1*.*2–5*.*8*	0.01	2.5	*0*.*96–6*.*3*	0.05	22	1.1	*0*.*32–3*.*4*	0.9	1.0	*0*.*28–3*.*9*	0.9	57	2.0	*1*.*1–3*.*9*	0.03	1.9	*0*.*9–4*.*0*	0.09
	BCL2 ≤ 85%	28							18							46						
	BCL2 > 70%	42	2.8	*1*.*1–6*.*8*	0.02	2.7	*0*.*9–7*.*9*	0.07	26	2.9	*0*.*62–13*	0.2	2.1	*0*.*44–10*	0.3	68	2.8	*1*.*3–6*.*1*	0.005	2.5	*1*.*0–6*.*1*	0.04
	BCL2 ≤ 70%	21							14							35						
	MYC > 75%	11	3.3	*1*.*5–7*.*3*	0.002	1.7	*0*.*61–4*.*5*	0.3	10	0.62	*0*.*14–2*.*9*	0.5	0.82	*0*.*17–3*.*9*	0.8	21	1.6	*0*.*82–3*.*2*	0.2	1.2	*0*.*52–2*.*8*	0.7
	MYC ≤ 75%	52							30							82						
	MYC > 40%	32	1.4	*0*.*71–2*.*9*	0.3	1.2	*0*.*51–2*.*7*	0.7	26	0.54	*0*.*16–1*.*8*	0.3	0.58	*0*.*16–2*.*2*	0.4	58	0.96	*0*.*52–1*.*7*	0.9	0.90	*0*.*44–1*.*8*	0.8
	MYC ≤ 40%	31							14							45						
	DE MYC > 40% + BCL2 > 70%	26	1.7	*0*.*85–3*.*4*	0.1	1.2	*0*.*54–2*.*9*	0.6	17	0.73	*0*.*21–2*.*5*	0.6	0.64	*0*.*16–2*.*6*	0.5	43	1.3	*0*.*72–2*.*4*	0.4	1.0	*0*.*50–2*.*1*	1.0
	DE MYC > 75% + BCL2 > 85%	10	4.4	*2*.*0–9*.*8*	<0.001	1.9	*0*.*72–5*.*3*	0.2	9	0.31	*0*.*040–2*.*4*	0.4	0.40	*0*.*050–3*.*2*	0.4	19	1.7	*0*.*83–3*.*4*	0.15	1.1	*0*.*47–2*.*8*	0.8
	GCB	30	1.0	*0*.*52–2*.*1*	0.9	1.2	*0*.*50–2*.*7*	0.7	24	2.0	*0*.*60–6*.*5*	0.3	2.1	*0*.*56–7*.*7*	0.3	54	1.3	*0*.*74–2*.*4*	0.3	1.5	*0*.*72–2*.*9*	0.3
	Non-GCB	33							16							49						
	GCB + DE MYC > 75% + BCL2 > 85%	6	8.7	*2*.*8–27*	<0.001	2.4	*0*.*61–9*.*2*	0.2	8	0.48	*0*.*054–4*.*3*	0.5	0.65	*0*.*067–6*.*2*	0.7	14	2.0	*0*.*79–5*.*0*	0.1	1.3	*0*.*40–4*.*0*	0.7
	GCB—DE MYC > 75% + BCL2 > 85%	24							16							40						
	Non-GCB + DE MYC > 75% + BCL2 > 85%	4	*	*	*	*	*	0.5	1	*	*	*	*	*	*	5	1.8	*0*.*53–6*.*0*	0,4	1.3	*0*.*31–5*.*9*	0.7
	Non-GCB—DE MYC > 75% + BCL2 > 85%	29							15							44						

R-CHOP, rituximab, cyclophosphamide, doxorubicin, vincristine, prednisone; R-CHOEP, rituximab, cyclophosphamide, doxorubicin, vincristine, etoposide, prednisone; PFS, progression free survival, OS, overall survival; HR, Hazard ratio; 95%CI, 95% confidence interval; F, female; M, male; aaIPI, age-adjusted international prognostic index; FISH, fluorescent in situ hybridization; IHC, immunohistochemistry; GCB, germinal center B-cell like.

* too few patients for meaningful statistical analyses.

### *MYC*, *BCL2* and *BCL6* translocation

FISH results are shown in Table 3. DH translocations were detected in 8/97 (8%) patients, most of whom (7/8, 88%) had GCB phenotype. *MYC*, *BCL2*, *BCL6* and DH translocation had no prognostic impact with respect to PFS or OS, neither in the treatment subgroups nor in the total cohort. DH translocations were too few to carry out meaningful statistical analyses in the subgroups of R-CHOP and R-CHOEP treated patients but unexpectedly, remarkably few events were seen (Table 3).

**Table 3:** Baseline molecular characteristics in all patients and in patients treated with R-CHOP and R-CHOEP.

		R-CHOP		R-CHOEP		TOTAL		p
		n	*%*	n	%	n	%	
FISH	*MYC +*	6	*10*	7	18	13	13	0.2
	*MYC -*	55	*87*	31	78	86	83	
	*MYC* unknown	2	*3*	2	5	4	4	
	*BCL6 +*	17	*27*	10	25	27	26	0.9
	*BCL6 -*	44	*70*	28	70	72		70
	*BCL6* unknown	2	*3*	2	5	4	4	
	*BCL2 +*	17	*27*	12	30	29	28	0.7
	*BCL2 -*	44	*70*	26	65	70	68	
	*BCL2* unknown	2	*3*	2	5	4	4	
	DH *MYC + BCL2/BCL6+*	3	*5*	5	13	8	8	**
IHC	BCL6 > 30%	50	*79*	36	90	86	83	0.15
	BCL6 ≤ 30%	11	*17*	3	8	14	14	
	BCL6 unknown	2	*3*	1	3	3	3	
	CD10 > 30%	24	*38*	18	45	42	41	0.5
	CD10 ≤ 30%	39	*62*	22	55	61	59	
	MUM1 > 30%	32	*51*	16	40	48	47	0.2
	MUM1 ≤ 30%	14	*22*	13	33	27	26	
	MUM1 unknown	17	*27*	11	28	28	27	
	GCB	30	*48*	24	60	54	52	0.2
	Non-GCB	33	*52*	16	40	49	48	
	BCL2 > 85%	35	*56*	22	55	57	55	1.0
	BCL2 ≤ 85%	28	*44*	18	45	46	45	
	BCL2 > 70%	42	*67*	26	65	68	66	0.9
	BCL2 ≤ 70%	21	*33*	14	35	35	34	
	MYC > 75%	11	*17*	10	25	21	20	0.4
	MYC ≤ 75%	52	*83*	30	75	82	80
	MYC > 40%	32	*51*	26	65	58	56	0.2
	MYC ≤ 40%	31	*49*	14	35	45	44	
	DE MYC > 75% + BCL2 > 85%		10	*16*	9	23	19	18	0.4
	DE MYC > 40% + BCL2 > 70%		26	*41*	17	43	43	42	0.9

R-CHOP, rituximab, cyclophosphamide, doxorubicin, vincristine, prednisone; R-CHOEP, rituximab, cyclophosphamide, doxorubicin, vincristine, etoposide, prednisone; FISH, fluorescent in situ hybridization; IHC, immunohistochemistry; GCB, germinal center B-cell like;

* *p* -Value: comparing R-CHOP to R-CHOEP;

** too few patients for meaningful statistical analyses.

### MYC and BCl2 protein expression

MYC and BCL2 protein expression had medians with IQRs on 50% (IQR 30%-70%) and 90% (IQR 50%-100%) respectively. The optimal cutoff values were MYC>75% and BCL2>85% (“Cutoff Finder”). The previously published lower cutoff levels MYC>40% and BCL2>70% were also studied for comparison. Distribution of IHC positive and negative patients with different cutoff values is listed in Table 3.

**Fig 1:**
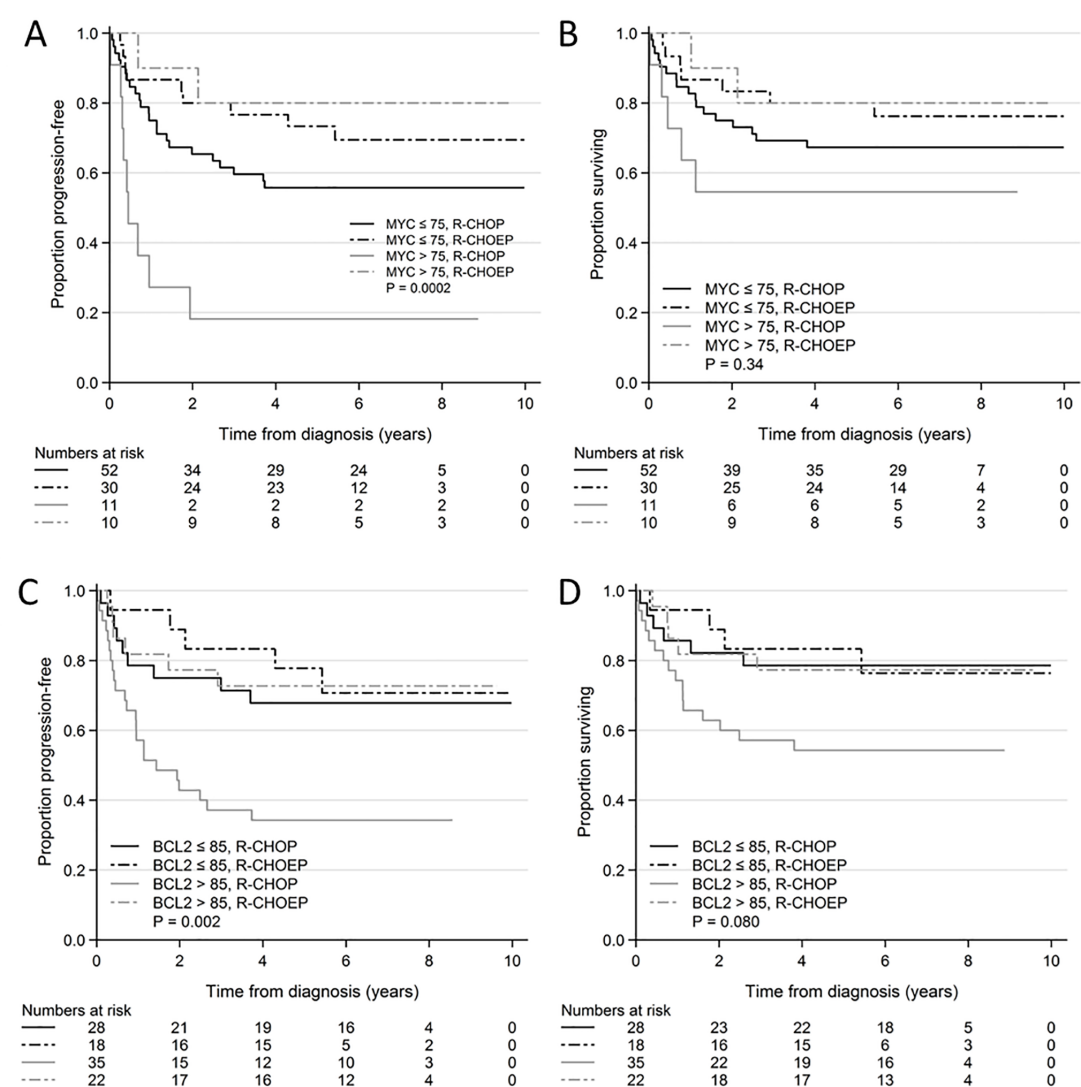
Kaplan Meier curves of MYC and BCL2 overexpression in R-CHOP and R-CHOEP treated patients. A: PFS, MYC>75% and MYC< = 75% in R-CHOP and R-CHOEP treated patients. B: OS, MYC>75% and MYC< = 75% in R-CHOP and R-CHOEP treated patients. C: PFS, BCL2>85% and BCL2< = 85% in R-CHOP and R-CHOEP treated patients. D: OS, BCL2>85% and BCL2< = 85% in R-CHOP and R-CHOEP treated patients. Abbreviations: PFS, progression free survival, R-CHOP, rituximab, cyclophosphamide, doxorubicin, vincristine, prednisone; R-CHOEP, rituximab, cyclophosphamide, doxorubicin, vincristine, etoposide, prednisone; OS, overall survival, P, p-value reflecting comparison of all 4 arms.

Patients with MYC>75% had significantly shorter PFS compared to patients with MYC≤75% when treated with R-CHOP. This was not seen in patients treated with R-CHOEP (Table 2, Fig 1)

The Kaplan Meier curves in Fig 1 show how both patients with MYC>75% and MYC≤75% seemed to benefit from R-CHOEP treatment when compared to R-CHOP. The difference in PFS between patients treated with R-CHOP and R-CHOEP was significant for patients with MYC> = 75% (p = 0.002). For patients with MYC<75% the difference was not significant (p = 0.2). With regards to OS the differences did not reach statistical significance (Table 2, Fig 1). No significant interactions between MYC>75% and treatment regimen were found (data not shown).

Patients with BCL2>85% had significantly lower PFS and OS compared to patients with BCL2≤85% when treated with R-CHOP. This prognostic effect was not seen in patients treated with R-CHOEP (Table 2, Fig 1). The difference in PFS and OS between patients treated with R-CHOP and R-CHOEP was significant for patients with BCL2> = 85% with regards to PFS (p = 0.007). Regarding OS the difference was not statistically significant (p = 0.09). For patients with BCL2<85% these differences were not significant (p = 0.7 and p = 1.0 respectively) (Fig 1). No statistically significant interactions were seen between BCL2>85 and treatment regimen (data not shown).

**Fig 2:**
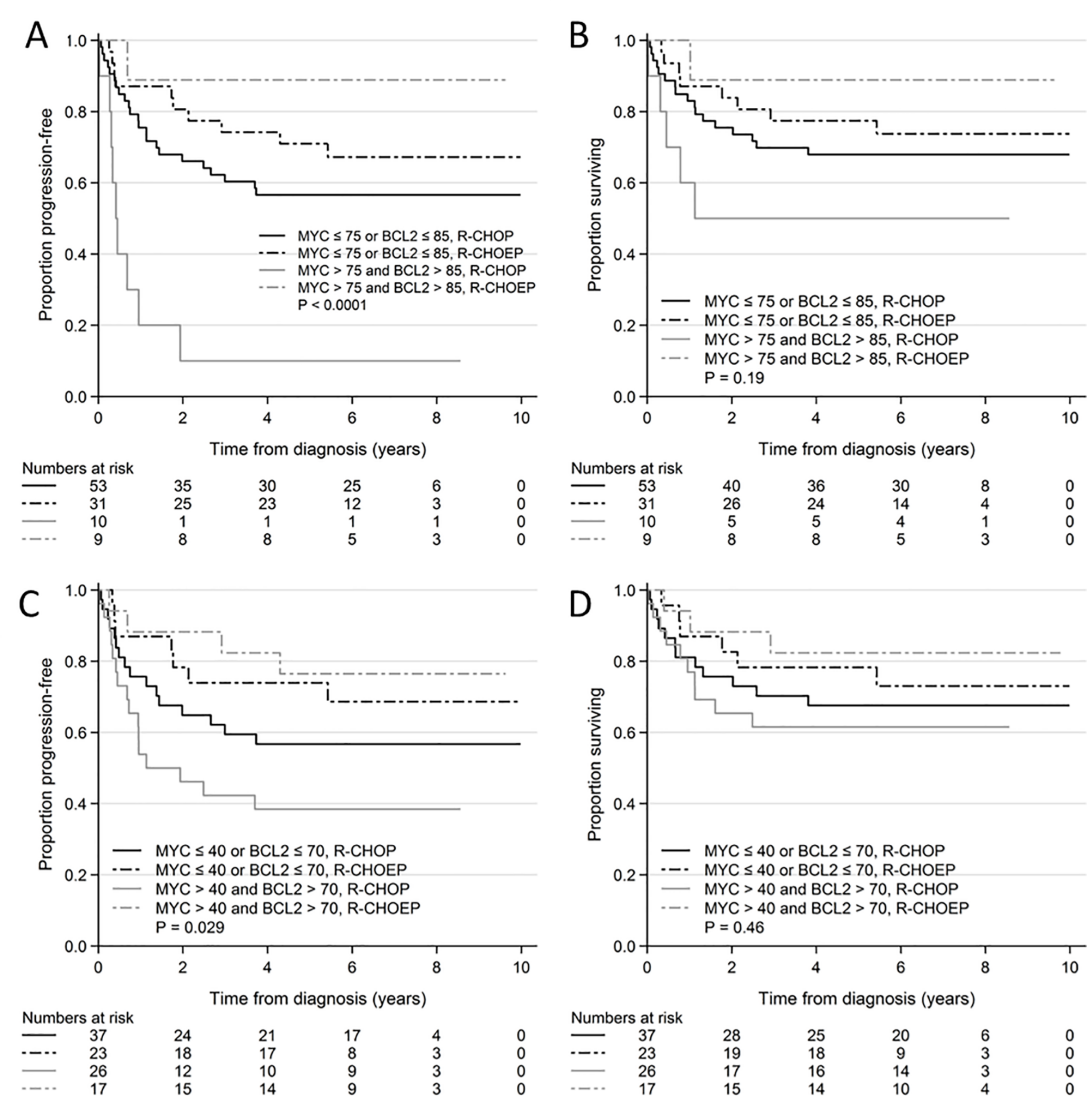
Kaplan Meier curves of MYC and BCL2 DE in R-CHOP and R-CHOEP treated patients with high and low cutoff values. A: PFS, DE (MYC>75% and BCL2>85%) and no DE in R-CHOP and R-CHOEP treated patients. B: OS, DE (MYC>75% and BCL2>85%) and no DE in R-CHOP and R-CHOEP treated patients. C: PFS, DE (MYC>40% and BCL2>70%) and no DE in R-CHOP and R-CHOEP treated patients. D: OS, DE (MYC>40% and BCL2>70%) and no DE in R-CHOP and R-CHOEP treated patients. Abbreviations: PFS, progression free survival, DE, double expression; R-CHOP, rituximab, cyclophosphamide, doxorubicin, vincristine, prednisone; R-CHOEP, rituximab, cyclophosphamide, doxorubicin, vincristine, etoposide, prednisone; OS, overall survival; P, p-value reflecting comparison of all 4 arms.

DE with MYC>75% and BCL2>85% was prognostic in R-CHOP treated patients with respect to PFS but not OS. In R-CHOEP treated patients DE had no significant prognostic effect with respect to either PFS or OS. The Kaplan Meier curves (Fig 2) show that both patients with and without DE seemed to benefit more from R-CHOEP when compared to R-CHOP. The difference in PFS between patients treated with R-CHOP and R-CHOEP was significant for patients with DE (p = 0.001) and the difference regarding OS was not statistically significant (p = 0.07). For patients without DE the differences were not significant (p = 0.3 and p = 0.5) (Table 2, Fig 2). In interaction analysis a possible statistical interaction was seen between DE (MYC>75% and BCL2>85%) and the treatment regimen for PFS (p = 0.07) but this was a non-statistically significant finding. No interaction was seen for OS (p = 0.2) (Table 2).

When the lower, previously published cutoff values of MYC>40% and BCL2>70% were applied for survival analysis overall the same results were seen with similar trends in the Kaplan Meier plots (Fig 2 and S1 Fig). The difference in PFS between patients treated with R-CHOP and R-CHOEP was significant for patients with BCL2>70% (p = 0.02). Regarding OS this difference was not statistically significant (p = 0.07). No differences were seen in patients treated with R-CHOEP. The differences no longer reached significant levels for MYC>40% and for DE of MYC>40% and BCL2>70% (Table 2). No statistical interactions were found (data not shown).

The survival analyses in which the prognostic impact from DE on PFS and OS was studied was repeated after exclusion of patients with DH translocations. The same tendencies were seen for both high cutoff values (DE of MYC>75% and BCL2>85%: PFS, R-CHOEP, p = 0.5; R-CHOP, p<0.001; OS, R-CHOEP, p = 0.6; R-CHOP, p = 0.1) and low cutoff values (DE of MYC>40% and BCL2>70%: PFS, R-CHOEP, p = 0.8; R-CHOP, p = 0.1; OS, R-CHOEP, p = 0.9; R-CHOP, p = 0.4).

With regard to response evaluation in the total cohort 4/21 (19%) patients with MYC>75% had no response to treatment (Stable disease (SD), progressive disease (PD), death) compared with 14/79 (18%) patients with MYC< = 75% (p = 0.9). There was also no difference after stratification according to treatment regimen (R-CHOP: 4/11 (36%) vs 10/50 (20%), p = 0.3; R-CHOEP: 0/10 (0%) vs 4/29 (14%), p = 0.6)

In the total cohort 14/57 (25%) patients with BCl2>85% had no response to treatment compared to 4/43 (9%) of patients with BCL2<85% (p = 0.07). After stratification according to treatment regimen a significant difference was seen in patients treated with R-CHOP (12/35 (34%) vs 2/26 (8%); p = 0.016) but not with R-CHOEP (2/22 (9%) vs 2/17 (12%); p = 1).

In the total cohort 4/19 (21%) patients with DE had no response to treatment compared to 14/81 (17%) patients without DE (p = 0.7). There was also no statistical difference after stratification according to treatment regimen (R-CHOP: 4/10 (40%) vs 10/51 (48%), p = 0.2; R-CHOEP: 0/9 (0%) vs 4/30 (13%) p = 0.6).

When the lower cutoff values of MYC>40% and BCL2>70% were applied no differences were seen between groups regarding response to treatment.

### Cell of origin

In this study COO was not a significant prognostic factor for PFS or OS in the total cohort, in R-CHOP or R-CHOEP treated patients (Table 2). The same tendency that GCB patients had greater benefit from R-CHOEP as previously described [15] was, however, seen in the Kaplan-Meier curves (S2 Fig).

COO results were previously published as a part of a larger population based study cohort in which it was shown that GCB patients had greater benefit from R-CHOEP compared to R-CHOP, with a statistically significant interaction detected between COO and treatment regimen [15].

### Cell of origin and DE

DE (MYC>75% and BCL2>85%) was more frequently found in patients with GCB phenotype (14/54, 26%) compared to patients with non-GCB phenotype (5/49, 10%) (p = 0.04). No interactions were found between COO and DE (MYC>75% and BCL2>85%). The prognostic effect of DE (MYC>75%and BCL2>85%) was studied in both patients with GCB and non-GCB phenotype treated with R-CHOP and R-CHOEP, respectively. Due to the small number of patients in these sub-groups the results should be considered with care. Similar findings were seen in patients with the GCB phenotype. Patients with non-GCB phenotype were too few in number for meaningful statistical analyses (Table 2).

### Multivariable analysis

A multivariable analysis was carried out in R-CHOP and R-CHOEP treated patients and included DE (MYC>75% + BCL2>85%) and aaIPI. DE was an independent significant prognostic marker of PFS in R-CHOP treated patients but not in R-CHOEP treated patients (R-CHOP: DE: HR 4.3; 95%CI: 1.9–9.6; p<0.001; R-CHOEP: DE: HR 0.32; 95%CI: 0.04–2.5; p = 0.3). With regard to aaIPI, a possible prognostic effect was seen in R-CHOP treated patients but not in R-CHOEP treated patients (R-CHOP: aaIPI: HR 2.0; 95%CI: 0.93–4.4; p = 0.08; R-CHOEP: aaIPI: HR 0.86; 95%CI: 0.19–4.0; p = 0.8).

With respect to OS, high aaIPI was a significant negative prognostic marker in patients treated with R-CHOP (HR 4.0 95%CI: 1.7–9.5; p = 0.002) but not R-CHOEP (HR 1.1 95%CI: 0.23–5.4; p = 0.9). DE had no statistically significant impact on OS in patients treated with either R-CHOP (HR 2.1; 95%CI: 0.75–5.6; p = 0.16) or R-CHOEP (HR 0.40; 95%CI: 0.05–3.2; p = 0.4).

## Discussion

Only young patients with high-risk DLBCL treated with either R-CHOP or R-CHOEP were included in this study. The cohort was of limited size, but it was unique and very uniform with regard to patients’ demographics such as age and treatment, allowing for a unique opportunity to study, albeit retrospectively, the effects of R-CHOEP compared to a control group of patients treated with R-CHOP and comparing these responses to established biomarkers such as COO, DE and DH.

We found that DE of MYC>75% and BCL2>85% had independent negative prognostic effect in patients treated with R-CHOP but not in patients treated with R-CHOEP. The same tendencies were seen using previously published cutoffs of MYC>40% and BCl2>70% but these findings were non-statistically significant. A possible interaction was found between DE and treatment regimen for PFS but this was also a non-statistically significant finding (p = 0.07). Based on this DE could be a possible predictive factor for treatment response with R-CHOEP compared to R-CHOP but this needs further investigation. A possible lack of treatment response was seen in patients with DE in R-CHOP treated patients but this was also a non-statistically significant finding. The lack of statistical significance in the analyses could possibly be explained by the limited cohort size. According to the 2016 WHO classification revision, patients with DH translocations should be excluded from the DLBCL NOS category and placed within the novel category “High-grade B-cell lymphomas, with *MYC* and *BCL2* or *BCL6* translocations”. We therefore repeated the survival analyses in which the prognostic impact from DE on PFS and OS was studied after exclusion of patients with DH translocations and similar findings were seen.

The results suggest that DE in young patients with high-risk DLBCL has prognostic importance in patients treated with R-CHOP as previously described in cohorts of patients with DLBCL with mixed clinical features, even though cutoff values in this study were different. By contrast, DE appeared to have minor prognostic impact in patients treated with R-CHOEP. The results also suggest that young patients with DE and high-risk DLBCL have improved outcome after treatment with R-CHOEP when compared to R-CHOP.

Whether DE and DH are prognostic factors in young patients with high-risk DLBCL has been but poorly investigated. In 2015 Horn et al. published a study of *MYC*, *BCL2* and *BCL6* translocations and protein-expression levels in approximately 100 young patients with high-risk DLBCL enrolled in the R-MegaCHOEP trial [22]. The patients were treated with either R-CHOEP or R-MegaCHOEP in a prospective randomized setting [9]. The study included no R-CHOP treated control group. A powerful prognostic effect of *BCL2* translocation was detected. *MYC* translocation, MYC protein expression and BCL2 protein expression only showed trends towards prognostic effects and DH did not indicate increased risk compared to single hit. The authors concluded that the prognostic value of DE appeared weaker in their cohort and suggested a potential variable importance of risk factors within clinically different patient cohorts and treatment groups [22]. The findings by Horn et al. are in accordance with the findings in the present study in which DE had prognostic value in R-CHOP but not in R-CHOEP treated young patients with high-risk DLBCL.

In the present study the log rank methodology was used to define the most optimal cutoff in R-CHOP treated patients using “Cutoff Finder” [20], introduced in order to standardize detection of cutoff values to reduce the risk of random findings. This methodology, however, has a risk of overestimating findings and therefore they should be validated in an independent patient cohort. Unfortunately this was not possible in the current study. However we also applied previously published cutoff values; MYC>40% and BCL2>70% [4;5] resulting in similar findings that were, however, not statistically significant. This limitation was possibly at least in part due to the relatively limited number of patients in the cohort, which implies that only powerful prognostic factors could be expected to be identified. We found both MYC and BCL2 protein expression higher than described in most studies. The prevalence of DE was 18% with the high cutoffs and 42% with the lower cutoffs whereas most studies described prevalence in between the two. This fact and a limited number of patients in the study makes the higher cut off values of MYC>75% and BCL2>85% seem reasonable in the current setting comparing different treatment regimens in small subgroups. Especially because the same tendencies were seen when the lower cut-offs values were applied. It should also be kept in mind that the patients included in this study all had high-risk DLBCL with a potential association with MYC and BCL2 levels.

Quantitative IHC for detection of MYC and BCL2 DE has proven difficult to introduce into clinical practice and different cutoff levels have been proposed by different institutions [23]. Inter-laboratory variation associated with IHC [24] and inter-observer variation especially regarding MYC scores in cases with heterogeneity or scores close to 40% [25;26] have been problematic. Standardization remains a precondition for the introduction of biomarkers into daily clinical practice.

In the current study a DH prevalence of 8% was found and 88% of these had GCB phenotype. This is in accordance with findings from previous studies. DH translocations had, however, no prognostic impact on PFS and OS and surprisingly few events (relapses/deaths) were observed in patients with these translocations. This could be due to the fact that only young patients with high-risk disease were included in the cohort but also to the limited number of patients in the cohort with DH translocation. DH translocations have previously predominantly been studied in cohorts with older patients [3–5;27–45].

We previously found that patients with GCB immunophenotype benefitted from R-CHOEP when compared to patients with non-GCB profile. This was not seen in R-CHOP treated patients and a statistically significant interaction between COO and treatment regimen was found [15]. In the current study cohort, we found the same tendencies. However, the results were not statistically significant, possibly because fewer patients were included (S2 Fig). In contrast with previously published data we found that DE was more frequently found among patients with GCB profile. This could possibly be explained by the different cutoff values used. This fact adds a possible bias to our previous findings that COO was predictive of response to treatment with R-CHOEP [15]. It was not possible to assess whether COO and DE were independent predictive markers in patients treated with R-CHOP. Further stratification of DE in patients with GCB and non-GCB profile respectively resulted in very small subgroups.

DA-EPOCH-R was previously suggested for patients with MYC and/or DH translocations based on preliminary data from a phase II study investigating DA-EPOCH-R in patients with *MYC* translocated aggressive lymphomas [11]. It was also suggested that especially patients with DLBCL and GCB profile or DE might benefit from DA-EPOCH-R [12;13]. Recently, preliminary data from a completed randomized phase III trial was published in which R-CHOP and DA-EPOCH-R were compared in patients with high-risk DLBCL, and surprisingly similar outcomes in the two treatment groups were reported [14]. Results from sub-stratification according to COO, DH translocation and DE in the randomized phase III trial with DA-EPOCH-R are currently awaited.

In this study we observed that both gender and aaIPI were prognostic in R-CHOP treated patients as expected, but this was not seen in patients treated with R-CHOEP when a very favorable outcome was seen (Table 3). Similarly, DE of MYC and BCL2 were not prognostic in patients treated with R-CHOEP. This underscores how well established prognostic markers in R-CHOP treated patients might have a more limited prognostic impact in patients treated with different, more aggressive chemotherapy regimens such as R-CHOEP associated with a very good outcome. This is of particular importance for future prospective trials with aggressive chemotherapy regimens for which patients are stratified based on these known prognostic markers. In order to conclude on the predictive value of a given marker, it is very important to demonstrate a statistically significant interaction between the biomarker investigated and the treatment regimen investigated [46].

In summary, we found that DE had a negative prognostic impact on outcome in this cohort of young patients with high-risk DLBCL, treated with R-CHOP but not in patients treated with R-CHOEP, suggesting that R-CHOEP could possibly overcome the negative prognostic impact of DE. We also observed a possible predictive value of DE for R-CHOEP compared to R-CHOP. Patients without DE had no significant benefit from R-CHOEP when compared to R-CHOP, but non-statistically significant differences were seen in the Kaplan Meier plots and therefore restriction of R-CHOEP for patients with DE cannot be supported by our data. DH translocations had no identifiable adverse prognostic impact in the current study, possibly also due to the limited number of patients. However, we cannot rule out the possibility that it could be explained by the younger age and/or the high-risk profile of patients. Although the patient cohort investigated in this study was of limited size the findings deserve further validation.

## Supporting information

S1 Fig Kaplan Meier curves of MYC>40% and BCL2>70% in R-CHOP and R-CHOEP treated patients. A: PFS, MYC>40% and MYC< = 40% in R-CHOP and R-CHOEP treated patients. B: OS, MYC>40% and MYC< = 40% in R-CHOP and R-CHOEP treated patients. C: PFS, BCL2>70% and BCL2< = 70% in R-CHOP and R-CHOEP treated patients. D: OS, BCL2>70% and BCL2< = 70% in R-CHOP and R-CHOEP treated patients.
Abbreviations: PFS, progression free survival, R-CHOP, rituximab, cyclophosphamide, doxorubicin, vincristine, prednisone; R-CHOEP, rituximab, cyclophosphamide, doxorubicin, vincristine, etoposide, prednisone; OS, overall survival, P, p-value reflecting comparison of all 4 arms.
(TIF)

S2 Fig Kaplan Meier curves of GCB and Non-GCB phenotype in R-CHOP and R-CHOEP treated patients. A: PFS, GCB and non-GCB in R-CHOP and R-CHOEP treated patients. B: OS, GCB and non-GCB in R-CHOP and R-CHOEP treated patients.
Abbreviations: PFS, progression free survival, GCB, germinal center B-cell like; R-CHOP, rituximab, cyclophosphamide, doxorubicin, vincristine, prednisone; R-CHOEP, rituximab, cyclophosphamide, doxorubicin, vincristine, etoposide, prednisone; OS, overall survival; P, p-value reflecting comparison of all 4 arms.
(TIF)
