# Supplementary figures and images for: Real world data on young patients with high-risk diffuse large B-cell lymphoma treated with R-CHOP or R-CHOEP - MYC, BCL2 and BCL6 as prognostic biomarkers
Source: PLoS One. 2017 Oct 31;12(10):e0186983. doi: 10.1371/journal.pone.0186983 (PMC5663399; doi:10.1371/journal.pone.0186983)

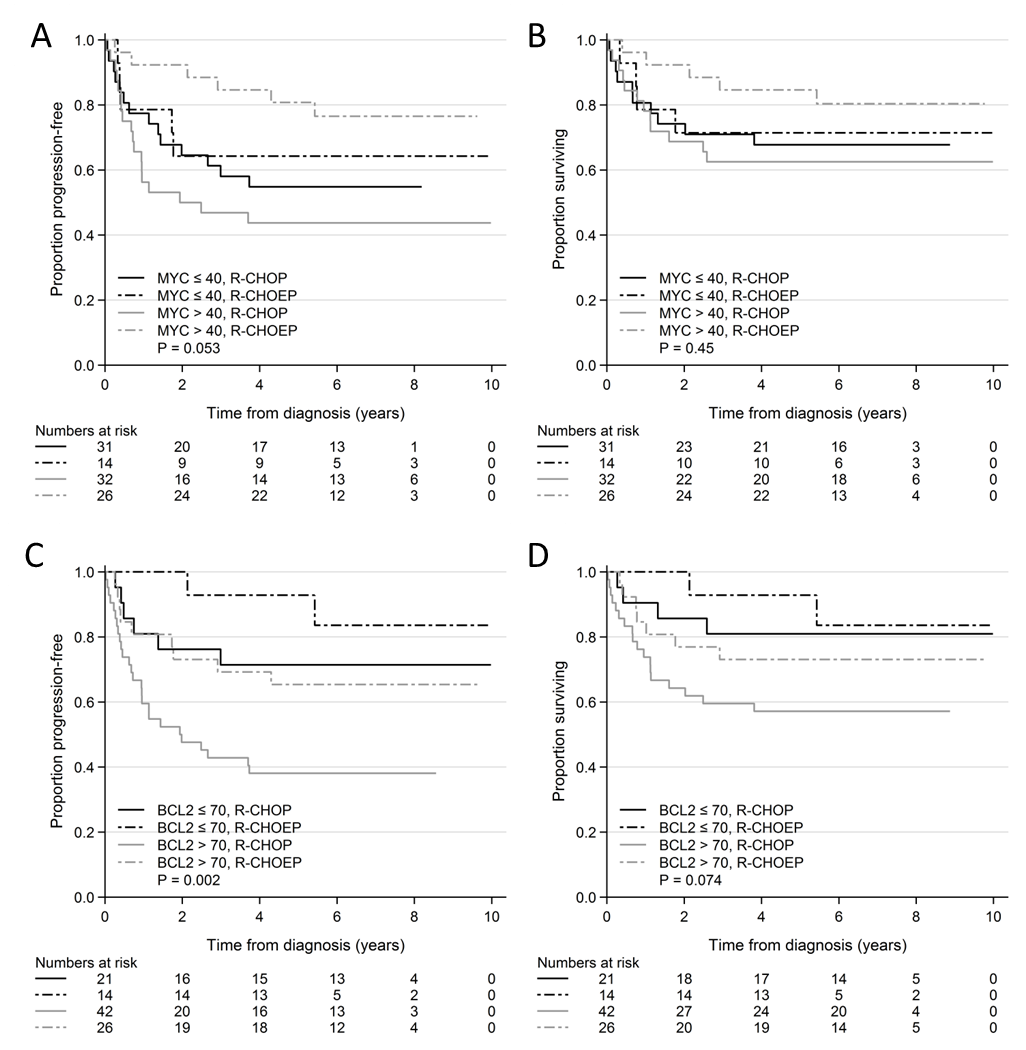

Supplement: S1 Fig — A: PFS, MYC>40% and MYC< = 40% in R-CHOP and R-CHOEP treated patients. B: OS, MYC>40% and MYC< = 40% in R-CHOP and R-CHOEP treated patients. C: PFS, BCL2>70% and BCL2< = 70% in R-CHOP and R-CHOEP treated patients. D: OS, BCL2>70% and BCL2< = 70% in R-CHOP and R-CHOEP treated patients. Abbreviations: PFS, progression free survival, R-CHOP, rituximab, cyclophosphamide, doxorubicin, vincristine, prednisone; R-CHOEP, rituximab, cyclophosphamide, doxorubicin, vincristine, etoposide, prednisone; OS, overall survival, P, p-value reflecting comparison of all 4 arms. (TIF) [file pone.0186983.s001.tif]

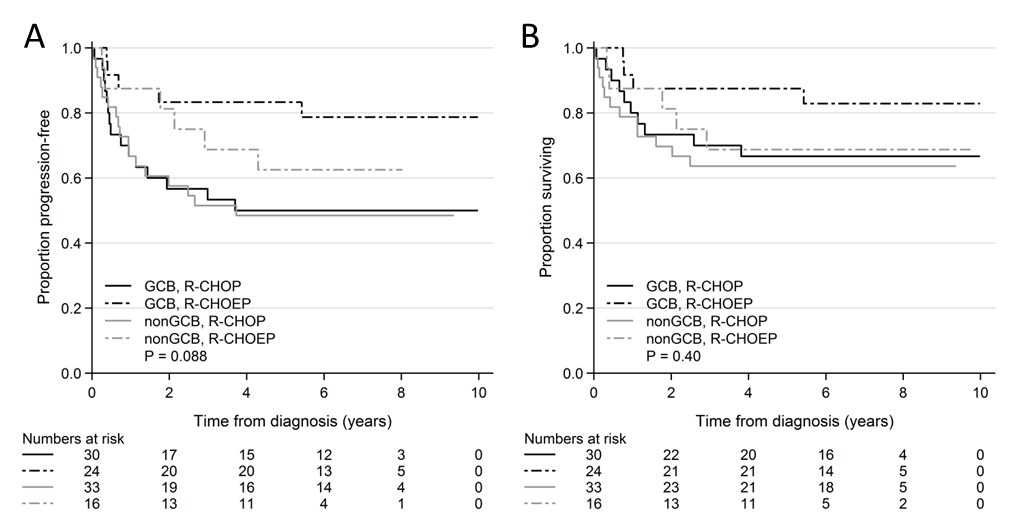

Supplement: S2 Fig — A: PFS, GCB and non-GCB in R-CHOP and R-CHOEP treated patients. B: OS, GCB and non-GCB in R-CHOP and R-CHOEP treated patients. Abbreviations: PFS, progression free survival, GCB, germinal center B-cell like; R-CHOP, rituximab, cyclophosphamide, doxorubicin, vincristine, prednisone; R-CHOEP, rituximab, cyclophosphamide, doxorubicin, vincristine, etoposide, prednisone; OS, overall survival; P, p-value reflecting comparison of all 4 arms. (TIF) [file pone.0186983.s002.tif]
